# Hypercompact TnpB and truncated TnpB systems enable efficient genome editing in vitro and in vivo

**DOI:** 10.1038/s41421-023-00645-w

**Published:** 2024-03-19

**Authors:** Ming Wang, Zhaolin Sun, Yue Liu, Pengbin Yin, Chuanyu Liang, Lin Tan, Lei Wei, Yuzhan Wang, Haikuan Yu, Yunfei Zhu, Xiaoxiang Hu, Ning Li, Ran Zhang

**Affiliations:** 1 State Key Laboratory of Animal Biotech Breeding, College of Biological Sciences, China Agricultural University, Beijing, China; 2 SciFriend Biotechnology Co., Ltd, Beijing, China; 3 Beijing Capital Agribusiness Future Biotechnology Co., Ltd, Beijing, China; 4 Department of Orthopaedics, Chinese PLA General Hospital, Beijing, China; 5 Frontiers Science Center for Molecular Design Breeding, China Agricultural University, Beijing, China

**Keywords:** Genomic analysis, Gene expression profiling

Dear Editor,

The bacterial adaptive immune systems CRISPR-Cas9/Cas12 have revolutionized genome editing in eukaryotic cells and in other various organisms^1^. Among the IS200/605 superfamily transposons, the IscB and TnpB proteins are likely progenitors of Cas9 and Cas12, respectively^2^. Recent studies have revealed functional similarities between these putative nucleases and Cas effector proteins, suggesting their programmable RNA-guided endonuclease activities^3,4^. Notably, the ISDra2-TnpB protein from *Deinococcus radiodurans* has demonstrated efficient DNA cleavage adjacent to the 5′-TTGAT transposon-associated motif (TAM) in HEK293T cells^4^. Moreover, with a compact size of only 408 amino acids, which is one-third of the size of the Cas9 protein, the ISDra2-TnpB system holds great promise for genome engineering applications due to its amenability to high-efficiency delivery^5,6^. However, certain important questions remain unanswered, such as the feasibility of using this compact ISDra2-TnpB system to generate gene-edited animals and implement gene therapy in vivo. Furthermore, the potential for streamlining and improving the compact ISDra2-TnpB system has not been fully explored. In this study, we present groundbreaking findings on the generation of mutant mice using ISDra2-TnpB and demonstrate its utility in in vivo adeno-associated virus (AAV)-based genome editing. Additionally, we introduce a truncated supermini TnpB editor (< 400 aa) generated by shortening the C-terminal domain (CTD) of TnpB, which also achieves efficient gene editing in mammalian cells and in mice.

To validate the editing activity of ISDra2-TnpB, we performed experiments in human cells (HEK293T and HeLa cells). Plasmids encoding the TnpB protein and right-element RNA (reRNA) were transiently transfected into the cells. After 72 h, DNA was analysed through deep sequencing to detect the insertions and deletions (indels) at the targeted sites (Supplementary Fig. S1a–c). In HEK293T cells, six tested sites (*EMX*-1, *AGBL1*-1, *AGBL1*-2, *ROSA26*-1, *ROSA26*-3 and *AAVS1*-2) showed efficient modifications (10 to 60%), while two tested sites (*ROSA26*-2 and *AAVS1*-1) exhibited moderate modification frequencies (1 to 5%) (Supplementary Fig. S1d). Analysis of the indels revealed that deletions were most prevalent at the cleavage site, with insertions and substitutions occurring less frequently (Supplementary Fig. S1e). These deletions were primarily small in size (< 50 bp), dominated by short deletions of 1–10 bp (Supplementary Fig. S1f, g). Importantly, most mutations were concentrated within 17–21 bp from the TAM (Supplementary Fig. S1h). ISDra2-TnpB showed lower editing activity in HeLa cells than in HEK293T cells, with a consistent editing pattern (Supplementary Fig. S2).

Then we evaluated the editing activity of ISDra2-TnpB in mouse NIH/3T3 cells. Fourteen 20-nt sites targeting eight genes (*Tyr*, *Rosa26*, *H11*, *Mstn, Tet1*, *Tet2*, *Tet3* and *PoLq*) were transfected into NIH/3T3 cells (Supplementary Fig. S3a). Deep sequencing analysis revealed varying mutation efficiencies across all sites, ranging from 2 to 50%, with deletions as the predominant indel type (Supplementary Fig. S3b, c). Short fragments of 1–10 bp were dominant, and again, mutations clustered within 17–21 bp from the TAM (Supplementary Fig. S3d–g).

Having demonstrated the high activity of ISDra2-TnpB in cultured cells, we proceeded with TnpB-mediated genome editing in mice. TnpB and reRNA targeting tyrosinase (*Tyr*) were microinjected into one-cell-stage embryos which were transplanted into surrogate mothers (Supplementary Fig. S4a). Analysis of *Tyr* exon1 using PCR and targeted deep sequencing revealed mutations at the cleavage site in 18% (9/50) of mice with 50 ng/μL DNA plasmid injection, 30.3% (10/31) with 100 ng/μL DNA plasmid injection, and 33.3% (12/36) with 100/50 ng/μL TnpB-mRNA/reRNA injection (Fig. 1a-c; Supplementary Fig. S4b, c). The *Tyr* mutant mice exhibited varying degrees of partial coat colour changes, consistent with their mosaic genotypes (Fig. 1a).

**Fig. 1:**
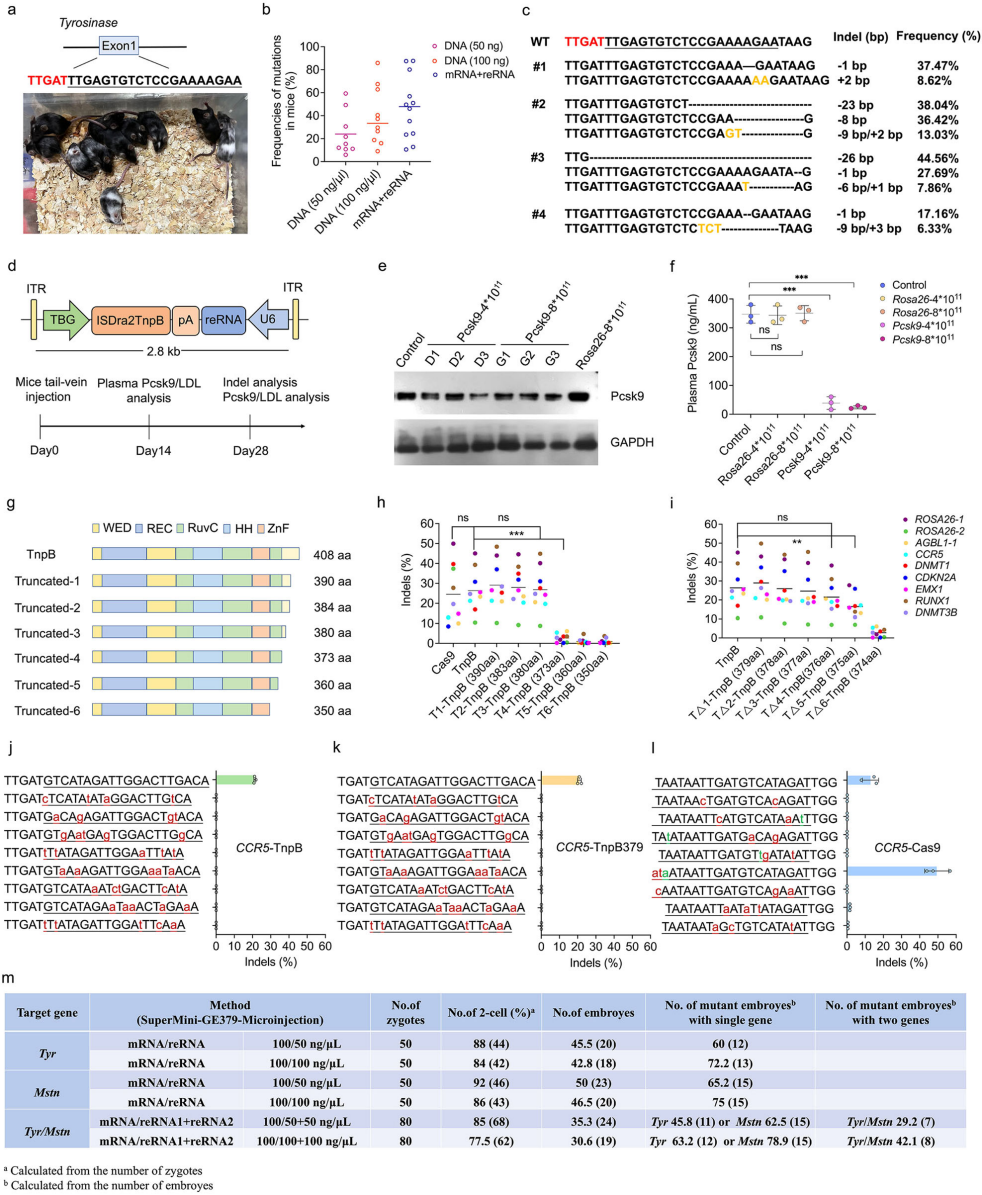
TnpB and truncated supermini TnpB-mediated genome editing in vitro and in vivo. **a** Representative images of *Tyr* mutant mice (F0) displaying various degrees of partial coat colour changes. **b** Indel frequencies in mice. PCR amplicons from the target regions in *Tyr* were analysed by targeted deep sequencing. Each dot represents one individual. **c** Targeted deep sequencing to analyse mutations at the target site. The TAM and target sequences are indicated in red and underlined, respectively. Microhomology sequences are shown in orange. **d** Workflow for in vivo delivery of AAV8 vectors in mice. Top: schematic representation of the all-in-one AAV vector expressing ISDra2-TnpB and reRNA. Bottom: timeline for AAV8 vector injection and subsequent analyses. **e** Western blotting analysis of plasma PCSK9 protein levels. **f** Plasma PCSK9 protein levels as determined by enzyme-linked immunosorbent assay (ELISA). Values represent the means ± SD of *n* = 3 animals. **g** Schematic representation of TnpB variants with C-terminal truncations. **h** Editing efficiencies of TnpB and its variants (TnpB390, TnpB383, TnpB380, TnpB373, TnpB360 and TnpB350) compared to that of Cas9 in HEK293T cells, as determined by deep sequencing. Each dot represents the average efficiency of three biological replicates. **i** Editing efficiencies of TnpB and its variants (TnpB373 to TnpB380) in HEK293T cells, as determined by deep sequencing. Each dot represents the average efficiency of three biological replicates. **j**–**l** Off-target effects of TnpB (**j**) and TnpB379 (**k**) and Cas9 (**l**) targeting the *CCR5* gene respectively. The underlined sequences represent the on-target or predicted off-target sites. The mismatches were labeled in red and lower case. The DNA bulges were labeled in green and lower case. Each point represents the editing efficiency of independent biological replicates by deep sequencing analysis. Data are presented as means ± SD, *n* = 3 independent biological replicates. **m** Summary of single-site or multiple-site gene editing by supermini TnpB379 in mouse embryos. One-way ANOVA with Geisser–Greenhouse correction was performed for statistics analyses. ns, not significant; *P < 0.05; **P < 0.01; ***P < 0.001.

The compact size of TnpB offers significant advantages for in vivo genome editing using AAV. To exploit this advantage, we generated an all-in-one AAV construct with TnpB expressed under the thyroxine-binding globulin (*TBG*) promoter and reRNA driven by the U6 promoter. In mice, two sites were targeted: the *Rosa26* gene (targeted by reRNA-*Rosa26*) as a negative control and the proprotein convertase subtilisin/kexin type 9 (*Pcsk*9) gene (targeted by reRNA-*Pcsk*9) as a phenotypic target. Tail vein injections were carried out, with two groups receiving 4 × 10^11^ AAV8 and two groups receiving 8 × 10^11^ AAV8. Serum samples were collected at 0, 14 and 28 days post-injection for LDL level measurement. At 28 days post-injection, the mice were sacrificed, and their liver tissues were harvested (Fig. 1d). Targeted deep sequencing revealed 9.5% and 22.9% average indel induction at the *Pcsk*9 and *Rosa26* editing sites in the liver, respectively, resulting in an ~90% reduction in plasma PCSK9 levels and a 50% reduction in plasma LDL levels (Fig. 1e, f; Supplementary Fig. S5a, b). To assess the specificity of TnpB in vivo, off-target analysis was conducted. For the *Pcsk*9 and *Rosa26* loci in mice, 5 predicted off-target sites were selected and no off-target effects were observed as shown by deep sequencing analysis (Supplementary Fig. S5c, d). Furthermore, we performed whole-genome sequencing (WGS) of hepatocytes from the AAV-treated animals. The results also showed no detectable off-target effects in the mice (Supplementary Fig. S5e). These results established the compact RNA-guided TnpB nuclease as an efficient and high-fidelity tool for genome editing.

To achieve a more concise structure, we further developed a truncated TnpB supermini editor with a shortened CTD domain. Initially, we designed six compact TnpB variants, namely TnpB390, TnpB383, TnpB380, TnpB373, TnpB360 and TnpB350 (Fig. 1g). These variants were transfected into HEK293T cells, and their editing efficiencies were evaluated at nine endogenous loci (*ROSA26*-1, *ROSA26*-2, *EMX1, AGBL1, CCR5, DNMT1*, *DNMT3B*, *CDKN2A* and *RUNX1*) using deep sequencing. The variants TnpB390 (29.6% ± 12.0%), TnpB383 (27.9% ± 10.7%) and TnpB380 (26.8% ± 10.9%), in which portions of the CTD domain were removed, exhibited editing efficiencies similar to that of TnpB (26.4% ± 10.7%). In contrast, TnpB373, TnpB360 and TnpB350, which lacked the entire CTD domain and parts of the RuvC domain, displayed significantly lower editing efficiencies (< 3.0% each). Notably, the editing efficiencies of TnpB and its variants surpassed that of Cas9 at six of nine tested loci (Fig. 1h). Further truncation optimization was carried out to create variant intermediates in length between TnpB373 and TnpB380. Analysis revealed that TnpB379 (29.0% ± 12.3%), TnpB378 (26.0% ± 11.9%), TnpB377 (24.6% ± 11.8%), TnpB376 (21.5% ± 9.2%) displayed editing efficiencies similar to that of TnpB (26.4% ± 10.7%) in all the tested sites. However, the editing efficiency of TnpB375 (16.5% ± 6.7%) was reduced by near half compared to that of TnpB (26.4% ± 10.7%) (Fig. 1i). We further evaluated the specificities of TnpB, TnpB379 and Cas9 targeting the *EMX1*, *AGBL*1, *ROSA26-1*, *DNMT1* and *CCR5* sites in HEK293T cells using Cas-OFFinder. Deep sequencing analysis of the 8 predicted off-target sites of each target revealed no obvious effects of TnpB or TnpB379 at any of the predicted off-target sites (Fig. 1j–l; Supplementary Fig. S6). Based on the above findings, we selected TnpB379 as the optimal supermini editor for further development.

Next, we evaluated the in vivo editing activity of the supermini-TnpB379 in two target genes, *Tyr* and *Mstn*. We co-injected in vitro transcribed reRNAs (*Tyr*-reRNA or *Mstn*-reRNA) with TnpB379-mRNA into 50 one-cell-stage embryos. For *Tyr*, targeted mutations were observed in 60% (12/20) and 72.2% (13/18) of embryos with TnpB379-mRNA/*Tyr*-reRNA injections at the concentrations of 100/50 ng/μL and 100/100 ng/μL, respectively (Fig. 1m; Supplementary Fig. S7). Similarly, for *Mstn*, targeted mutations were observed in 65.2% (15/23) and 75.0% (15/20) of embryos with TnpB379-mRNA/*Mstn*-reRNA injections at the concentrations of 100/50 ng/μL and 100/100 ng/μL, respectively (Fig. 1m; Supplementary Fig. S8). To confirm the potential for multiple-site editing with supermini-TnpB379, we co-injected *Tyr*-reRNA and *Mstn*-reRNA with TnpB379-mRNA into 80 one-cell-stage embryos. With a 100/50/50 ng/μL TnpB379-mRNA/*Tyr*-reRNA/*Mstn*-reRNA injection, targeted mutations were observed in 45.8% (11/24) and 62.5% (15/24) of embryos for *Tyr* and *Mstn*, respectively. Among the screened blastocysts, 29.2% (7/24) of embryos had targeted mutations in both genes. With 100/100/100 ng/μL TnpB379-mRNA/*Tyr*-reRNA/*Mstn*-reRNA injection, targeted mutations were observed in 63.2% (12/19) and 78.9% (15/19) of embryos for *Tyr* and *Mstn*, respectively, and 42.1% (8/19) of the screened blastocysts had targeted mutations in both genes (Fig. 1m; Supplementary Fig. S9).

In summary, our study highlights the remarkable potential of ISDra2-TnpB as a highly efficient tool for achieving site-specific modifications both in vitro and in vivo. Moreover, we successfully generated a novel truncated supermini ISDra2-TnpB (< 400 aa), which also exhibits efficient genome editing capabilities in mammalian cells and mice. The extremely compact non-Cas nuclease TnpB and the truncated supermini ISDra2-TnpB represent a promising and versatile tool for diverse genome editing applications.

### Supplementary information

Supplementary information

## References

[1] Hille F (2018). Cell.

[2] Makarova KS (2020). Nat. Rev. Microbiol..

[3] Altae-Tran H (2021). Science.

[4] Karvelis T (2021). Nature.

[5] Xu X (2021). Mol. Cell.

[6] Kim DY (2022). Nat. Biotechnol..

