# Supplementary material for: Hypercompact TnpB and truncated TnpB systems enable efficient genome editing in vitro and in vivo
Source: Cell Discov. 2024 Mar 19;10:31. doi: 10.1038/s41421-023-00645-w (PMC10951260; doi:10.1038/s41421-023-00645-w)
Supplement: Supplementary file 1 — Supplementary information [file 41421_2023_645_MOESM1_ESM.pdf]

Hypercompact TnpB and truncated TnpB systems enable efficient genome editing in vitro and in vivo

Ming Wang[1,2], Zhaolin Sun[3], Yue Liu[1], Pengbin Yin[4], Chuanyu Liang[1], Lin Tan[1], Lei Wei[1], Yuzhan Wang[1], Haikuan Yu[4], Yunfei Zhu[1], Xiaoxiang Hu[1], Ning Li[3], Ran Zhang[1, 5]

1. State Key Laboratory of Animal Biotech Breeding, College of Biological Sciences, China Agricultural University, Beijing, China.
2. SciFriend Biotechnology Co., Ltd, Beijing, China.
3. Beijing Capital Agribusiness Future Biotechnology Co., Ltd, Beijing, China.
4. Department of Orthopaedics, Chinese PLA General Hospital, Beijing, China.
5. Frontiers Science Center for Molecular Design Breeding, China Agricultural University, Beijing, China.

These authors contributed equally to this work: Ming Wang, Zhaolin Sun

Correspondence: Ran Zhang (zhangran0628@cau.edu.cn) or Ning Li (lining@bjsngf.com.cn).

Supplementary Figures

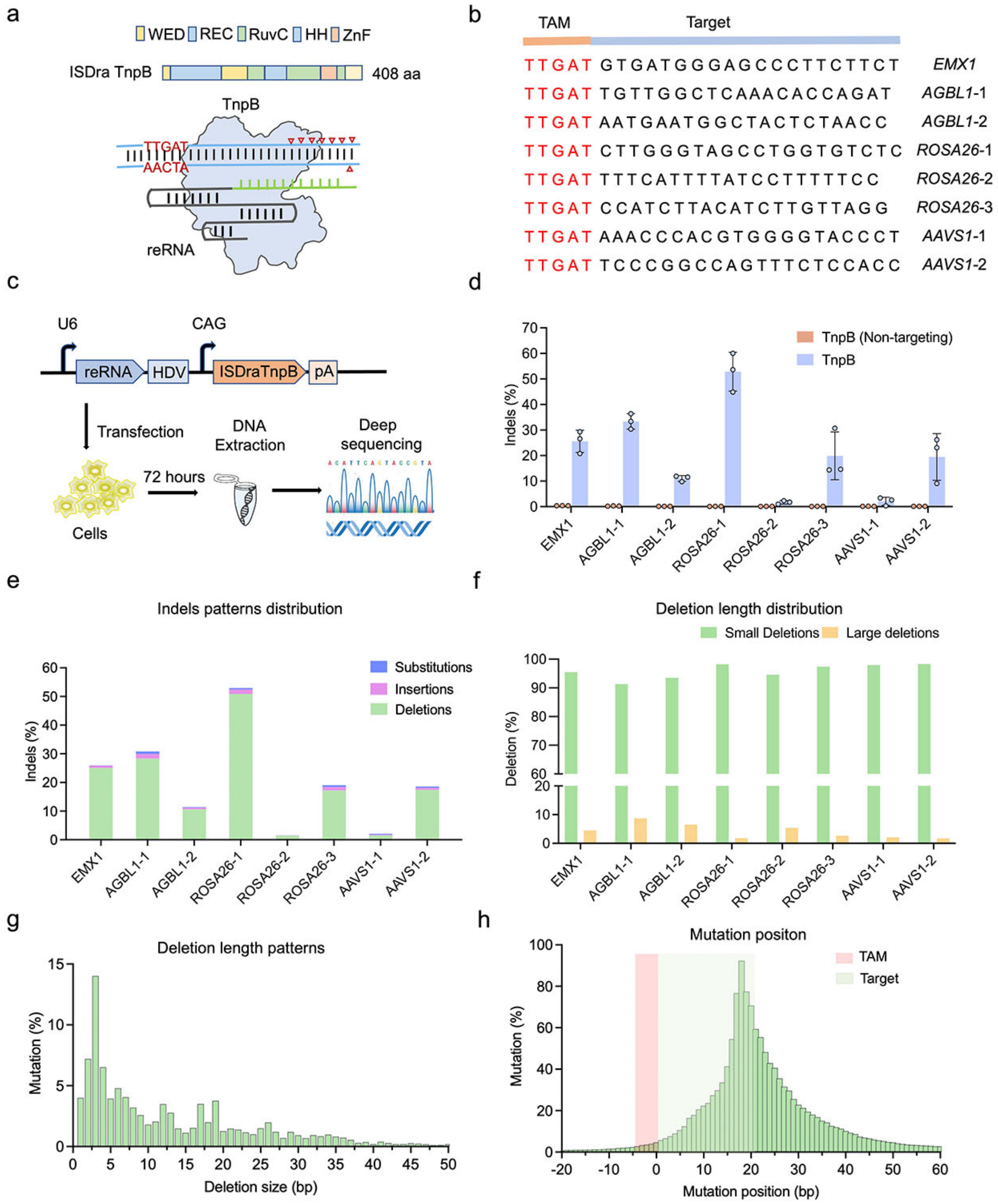

Supplementary Figure 1. TnpB nuclease-mediated gene editing in HEK293T cells.

(a) Schematic representation of TnpB nuclease structural domains/motifs and gene editing mechanism. WED: wedge domain, REC: helical bundle, RuvC: RuvC domain, HH: helical hairpin, ZnF: zinc-finger domain. **(b)** Detailed reRNA sequence information for the 8 target sites with TAM sequences for TnpB (highlighted in orange). **(c)** Experimental workflow of the human cell line (HEK293T) genome editing experiment. Plasmids carrying both TnpB and reRNA were transfected into HEK293T cells, and the transfected cells were analysed at 72 h

posttransfection, followed by deep sequencing. **(d)** Detection of indel activity at the 8 tested 20-nt targets in human HEK293T genomic DNA. Data represent the mean of three biologically independent experiments (shown as dots) ± standard deviation). The TnpB (Non-targeting) expression plasmid encodes reRNA containing a guide sequence and was used as a negative control. **(e)** Distribution of targeted TnpB-mediated indel patterns at the 8 target loci in HEK293T cells. Each target contains three biological replicates, and the data are presented as the mean ± SD. **(f)** Analysis of the percentages of small deletions (≤50 bp, in green) and large deletions (>50 bp, in yellow) at the 8 target loci in HEK293T cells. Each target contains three biological replicates, and the data are presented as the mean ± SD). **(g)** Size and positional information for deletions within 50 bp of the target site generated by the indicated TnpB nucleases at all tested sites in HEK293T cells. The vertical axis indicates the average ratio of the number of deletion fragments with the indicated length to the total number of deletion events. n=8, and each target contains three biological replicates. **(h)** Analysis of deletion positions in TnpB-mediated gene editing in HEK293T cells. The TAM and 20-bp target are shown in light red and green. n=8, and each target contains three biological replicates.

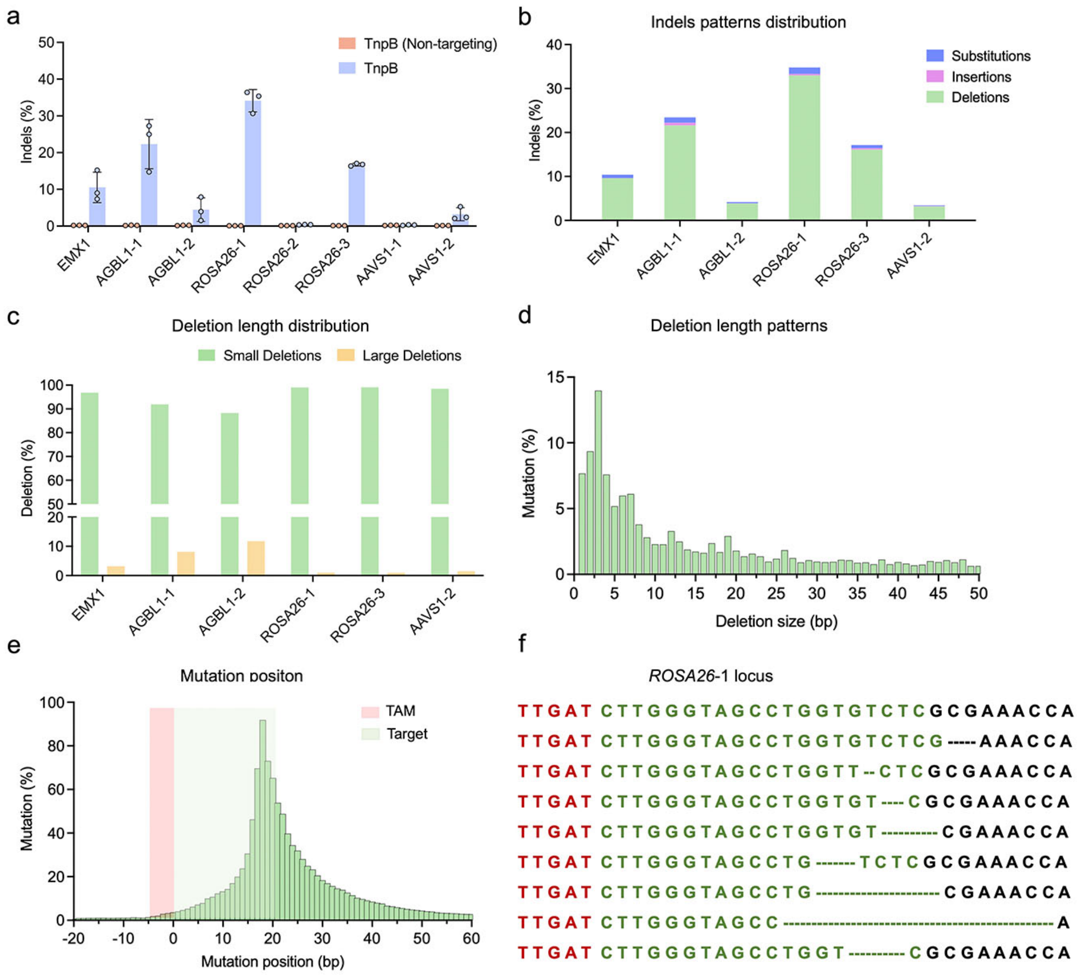

Supplementary Figure 2. TnpB nuclease-mediated gene editing in HeLa cells.

(a) Detection of indel activity at the 8 tested 20-nt targets in human HeLa genomic DNA. Data represent the mean of three biologically independent experiments (shown in dots) ± standard deviation). The TnpB (Non-targeting) expression plasmid encodes reRNA containing a guide sequence and was used as a negative control. **(b)** Distribution of targeted TnpB-mediated indel patterns in HeLa cells. Each target contains three biological replicates, and the data are presented as the mean ± SD. **(c)** Analysis of the percentages of small deletions (≤50 bp, in green) and large deletions (>50 bp, in yellow) at 6 loci in HeLa cells. Each target contains three biological replicates, and the data are presented as the mean ± SD. **(d)** Size and positional information for deletions within 50 bp of the target site generated by the indicated TnpB nucleases at all tested sites in HeLa cells. The vertical axis indicates the average ratio of the number of deletion fragments with the indicated length to the total number of deletion events. n=6, and each target contains three biological replicates. **(e)** Analysis of deletion position in TnpB-mediated gene editing in HeLa cells. The TAM and 20-bp target are shown in light red

and green, respectively. n=6, and each target contains three biological replicates. **(f)** Representative indel information at the *ROSA26*-1 locus in HeLa cells. The TAM and 20-bp target are shown in red and green, respectively.

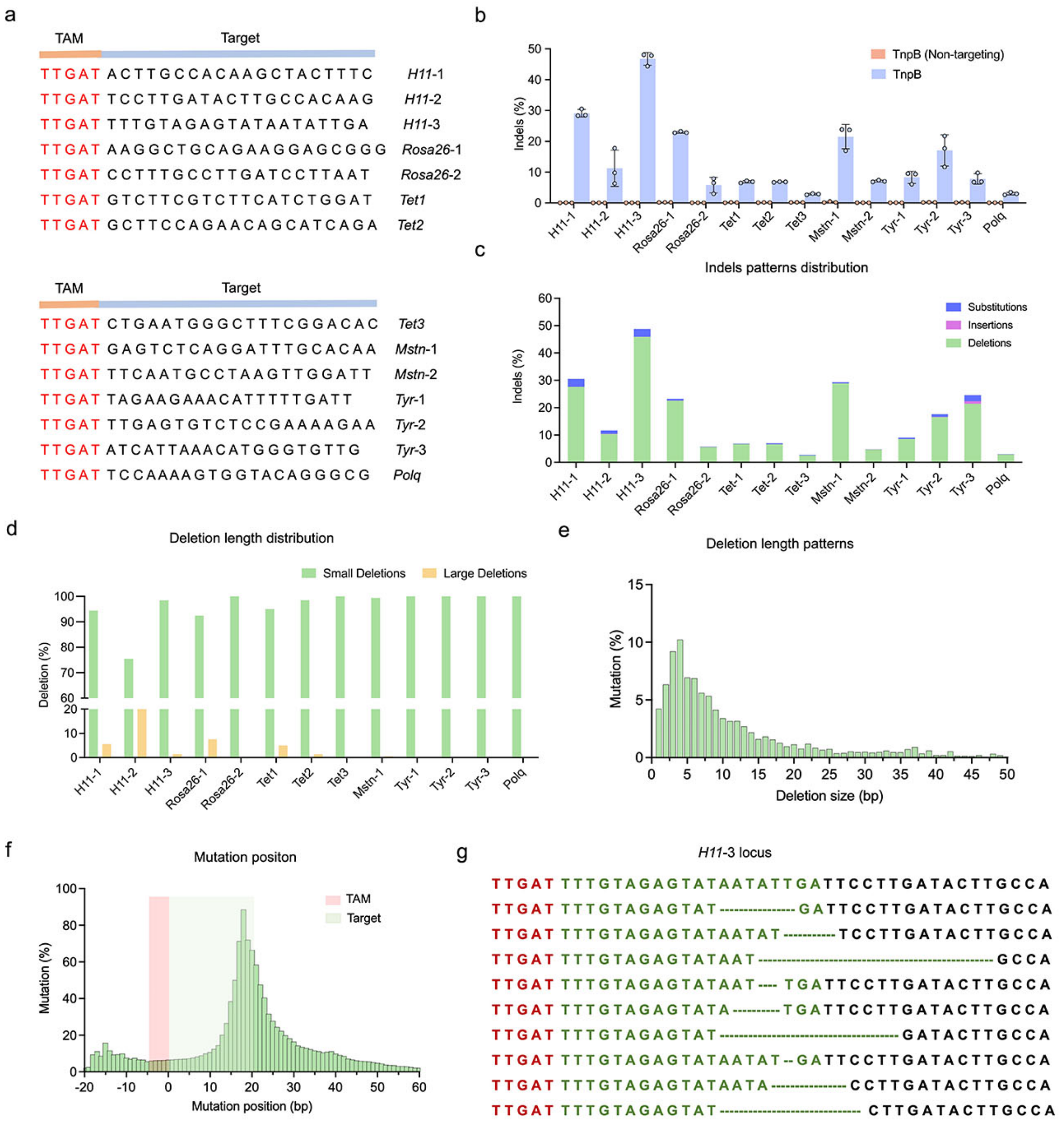

Supplementary Figure 3. TnpB nuclease-mediated gene editing in mouse NIH/3T3 cells. **(a)** Detailed reRNA sequence information of the 14 target sites with TAM sequences for TnpB in orange. **(b)** Detection of indel activity at the 14 tested 20 nt length targets in mouse NIH/3T3 genomic DNA. Data represent the mean of three biologically independent experiments (shown in dots) ± standard deviation). The TnpB (Non-targeting) expression plasmid encodes reRNA containing a guide sequence and was used as a negative control. **(c)** Distribution of targeted TnpB-mediated indel patterns at 14 target loci in NIH/3T3 cells. Each target contains three biological replicates, and the data are presented as the mean ± SD. **(d)** Analysis of percentages of small deletions (≤50 bp, in green) and large deletions (>50 bp, in yellow) at 13 loci in NIH/3T3 cells. Each target contains three biological replicates, and the data are presented as

the mean ± SD. **(e)** Size and positional information for deletions within 50 bp of the target site generated by the indicated TnpB nucleases at all tested sites in NIH/3T3 cells. The vertical axis indicates the average ratio of the number of deletion fragments with the indicated length to the total number of deletion events. n=14, and each target contains three biological replicates. **(f)** Analysis of deletion position in TnpB-mediated gene editing in NIH/3T3 cells. The TAM and 20-bp target are shown in light red and green. n=14, and each target contains three biological replicates. **(g)** Representative indel information at the *H11*-1 locus in NIH/3T3 cells. The TAM and 20-bp target are shown in red and green, respectively.

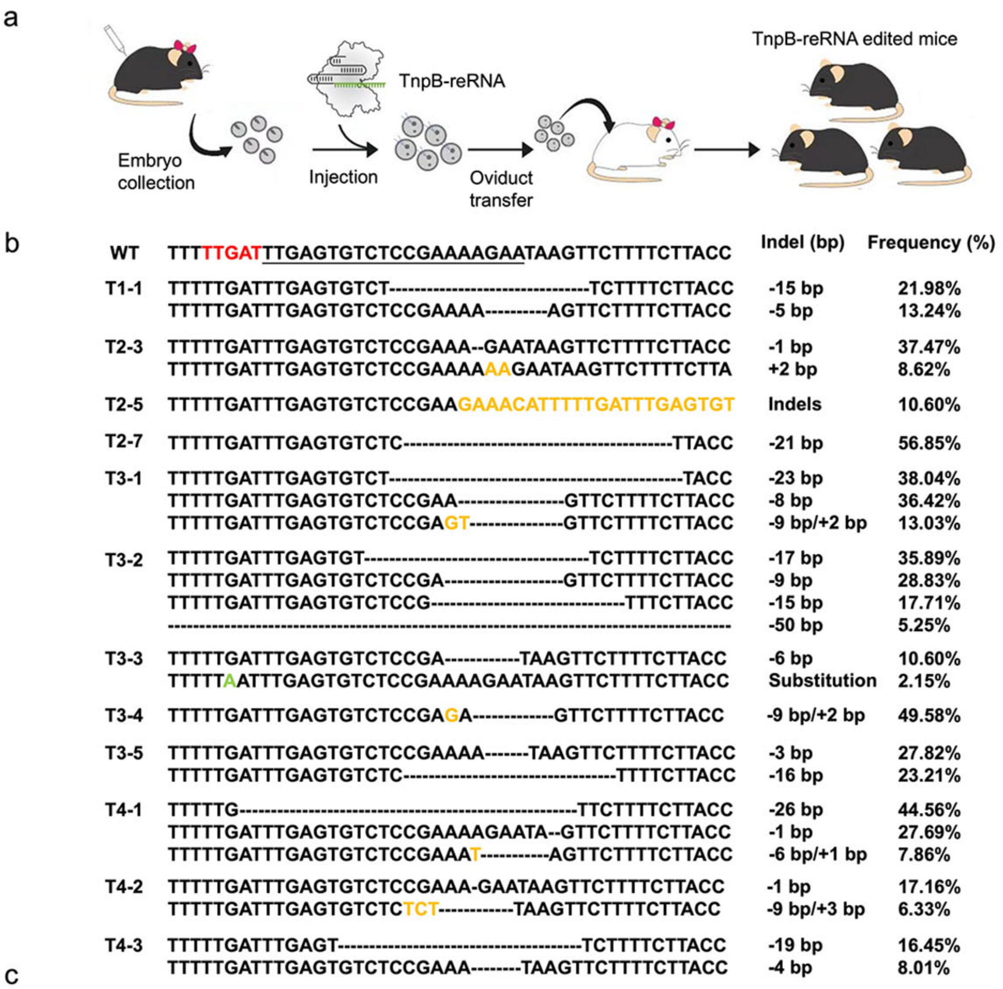

c

Target gene	Method		No.of zygotes	No.of 2-cell (%)[a]	No.of Recipients	No. of offspring	No. of mutant offspring[b]
Tyr	DNA (50 ng/μL)	Microinjection	100	90 (90)	4	50	9 (18)
	DNA (100 ng/μL)	Microinjection	100	86 (86)	3	31	10 (30.3)
	mRNA+reRNA	Microinjection	100	88 (88)	4	36	12 (33.3)

[a] Calculated from the number of zygotes
[b] Calculated from the number of offspring

Supplementary Figure 4. TnpB-mediated genome editing in mice by microinjection.

(a) Graphical overview of TnpB-mediated genome editing in mice by microinjection. Approximately 4-week-old female mice are superovulated and mated with males for breeding. Pronuclear-stage embryos are collected and subjected to microinjection, with TnpB /reRNA complexes assembled *in vitro*. The microinjected embryos are then transferred to pseudopregnant surrogate mothers to generate genetically engineered mice, which are subsequently genotyped to confirm editing efficiency. **(b)** Targeted deep sequencing to analyse mutations at the target site. The TAM and target sequences are indicated in red and underlined,

respectively. Microhomology sequences are shown in orange, and base substitutions are shown in green. **(c)** Summary of the numbers of embryos used and mutants generated by TnpB-reRNA targeting of the *Tyr* gene.

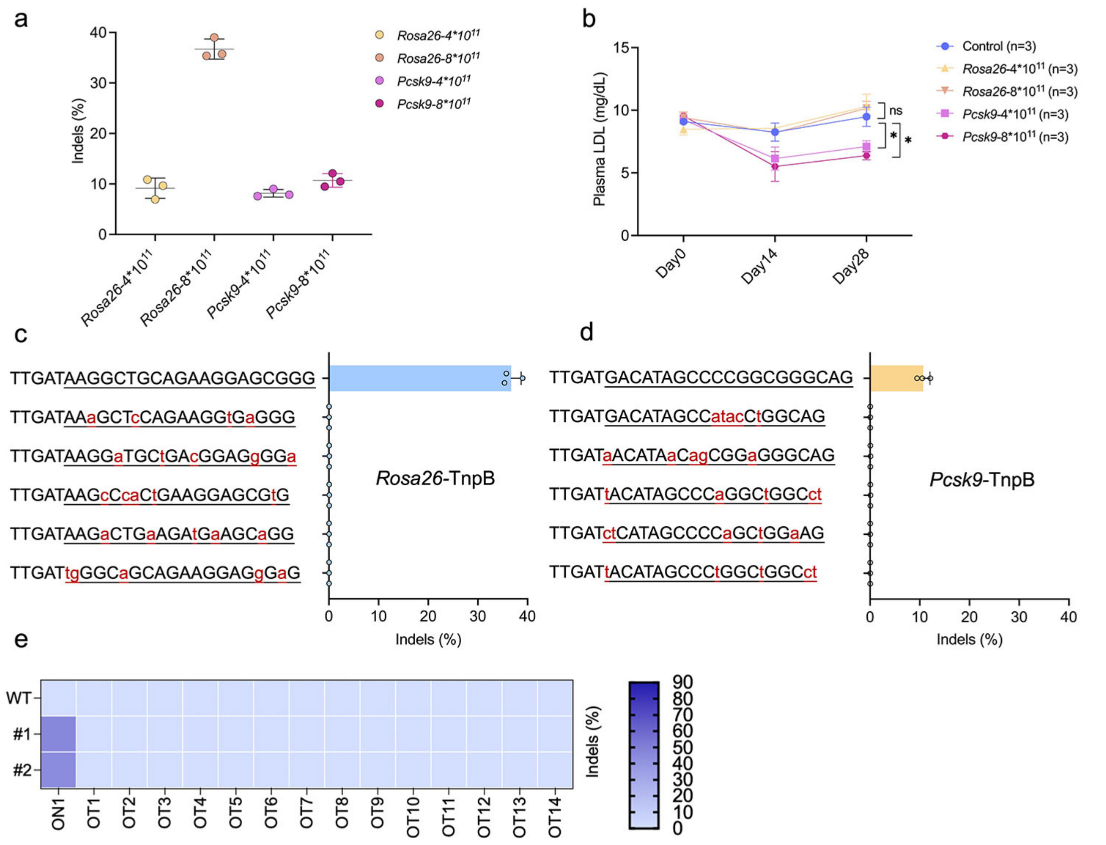

Supplementary Figure 5. TnpB genome editing *in vivo* via all-in-one AAV delivery

(a) Indels in DNA extracted from livers of mice injected with AAV8.reRNA.ISDra2-TnpB targeting *Pcsk9* and *Rosa26*. Values represent mean ± SD of n = 3 animals. **(b)** Plasma LDL cholesterol levels. Values represent the mean ± SD of n = 3 animals. **(c-d)** Off-target effects of TnpB targeting *Rosa26* (c) and *Pcsk9* (d) gene respectively. The underlined sequences represent the on-target or predicted off-target sites. The mismatches were labeled in red and lower case. Each point represents the editing efficiency of independent animal by deep sequencing analysis. Data are presented as means ± SD, n = 3. **(e)** Off-target effects of TnpB targeting *Rosa26* gene by whole-genome sequencing. WT, wild type mice; #1- #2, AAV-treated mice.

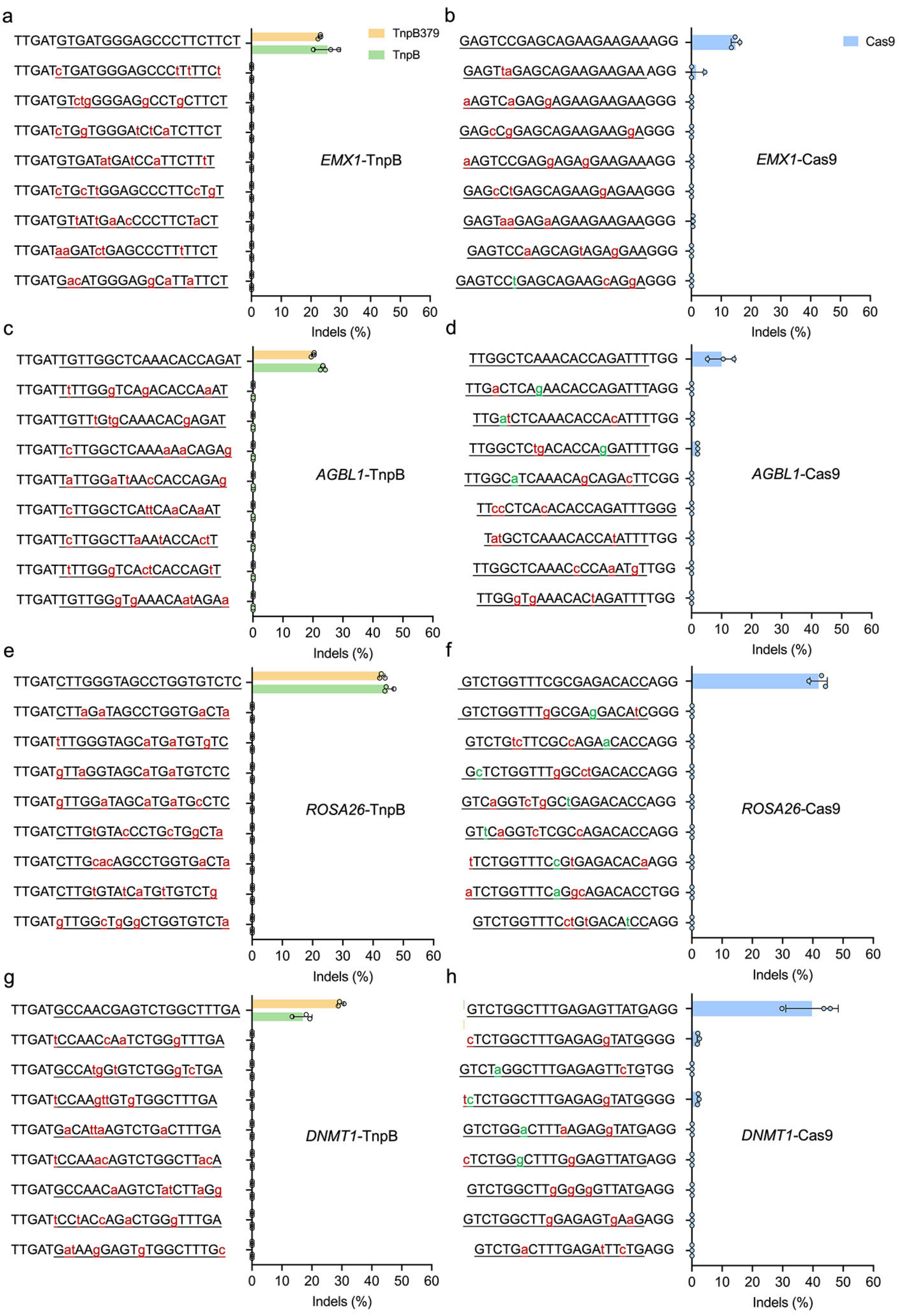

Supplementary Figure 6. Off-target efficiency of TnpB, TnpB379 and Cas9 at predicted off-target sites of *EMX1*, *AGBL*1, *ROSA26-1* and *DNMT1* genes.

(a)-(b) Off-target effects of TnpB and TnpB379 (a) and Cas9 (b) targeting *EMX1* gene respectively. **(c)-(d)** Off-target effects of TnpB and TnpB379 (c) and Cas9 (d) targeting *AGBL1*

gene respectively. **(e)-(f)** Off-target effects of TnpB and TnpB379 (e) and Cas9 (f) targeting *ROSA26* gene respectively. **(g)-(h)** Off-target effects of TnpB and TnpB379 (g) and Cas9 (h) targeting *DNMT1* gene respectively. The underlined sequences represent the on-target or predicted off-target sites. The mismatches were labeled in red and lower case. The DNA bulge were labeled in green and lower case. Each point represents the editing efficiency of independent biological replicate by deep sequencing analysis. Data are presented as means ± SD, n = 3 independent biological replicates.

a

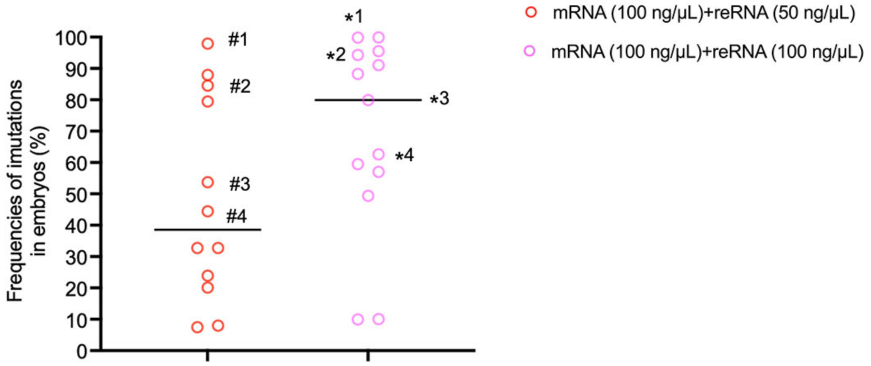

b

	Sequence	Indel (bp)	Frequency (%)
WT	TTT<u>TTGAT</u>TTGAGTGTCTCCGAAAAGAATAAGTTCTTTTCTTACC		
#1	TTTTTGATTTGAGT--------------------------------------TCTTTTCTTACC	-19 bp	42.13%
	TTTTTGATTTGAGTGTCTC-------------------------------- TTTTCTTACC	-16 bp	35.65%
	TTTTTGATTTGAGTGTCTCCGACA--------------- TTCTTTTCTTACC	-10 bp/+2 bp	20.78%
#2	TTTTTGATTTGAGTGTCT--- TACC	-23 bp	53.15%
	TTTTTG--- TTTTCTTACC	-29 bp	30.16%
#3	TTTTTGATTTGAGTGTCTCCGAAA-- GAATAAGTTCTTTTCTTACC	-1 bp	38.57%
	TTTTTGATTTGAGTGTCT------------------------------------TTTCTTACC	-18 bp	13.60%
#4	TTTTTGATTTGAGTGTCTC--------------------------------TTTTCTTACC	-16 bp	43.44%
*1	TTTTTGATTTGAGTGTCTC---	-28 bp	66.08%
	TTTTTGATTTGAGTGTCTC ------------------------------ TTTTCTTACC	-16 bp	31.75%
	TTTTTGATTTGAGTGTCT------------------------------------ TTTCTTACC	-18 bp	1.07%
*2	TTTTTGATTTGAGTGTCTC ------------------------------ TTTTCTTACC	-28 bp	47.70%
	TTTTTGATTTGAGT--------------------------------------TCTTTTCTTACC	-19 bp	45.54%
*3	TTTTTGATTTGAGTGT-----------------------------------TCTTTTCTTACC	-17 bp	48.60%
	TTTTTGATTTGAGTGTCTCCGA------------------GTTCTTTTCTTACC	-9bp	12.11%
	TTTTTGATTTGAGTGTCTCCGGG----------------------- TTTTCTTACC	-14 bp/+2 bp	10.05%
	TTTTTGATTTGAGTGTCTCCGAA-----GAATAAGTTCTTTTCTTACC	-2 bp	7.72%
	TTTTTGATTTGAGTGTCTCCGAAGA----------AGTTCTTTTCTTACC	-7 bp/+2 bp	4.36%
	TTTTTGATTTGAGTGTCT------------------------------------TTTCTTACC	-18 bp	3.65%
*4	TTTTTGATTTGAGTGTCTCCGAAA---GAATAAGTTCTTTTCTTACC	-1 bp	42.90%
	TTTTTGATTTGAGTGTCT------------------------------------TTTCTTACC	-18 bp	9.56%
	TTTTTGATTTGAGTGTCTC--------------------------------TTTTCTTACC	-16 bp	9.16%
	TTTTTGATTTGAGTGTCTCCG---------GAATAAGTTCTTTTCTTACC	-4 bp	2.39%

Supplementary Figure 7. TnpB379-mediated genome editing in mouse *Tyr* by microinjection.

(a) Indel frequency in mouse embryos generated by microinjection. PCR amplicons from the target regions in *Tyr* were analysed by targeted deep sequencing. Each dot represents one individual. **(b)** Targeted deep sequencing to analyse mutations at the target site. The TAM and target sequences are indicated in red and underlined, respectively. Microhomology sequences are shown in orange.

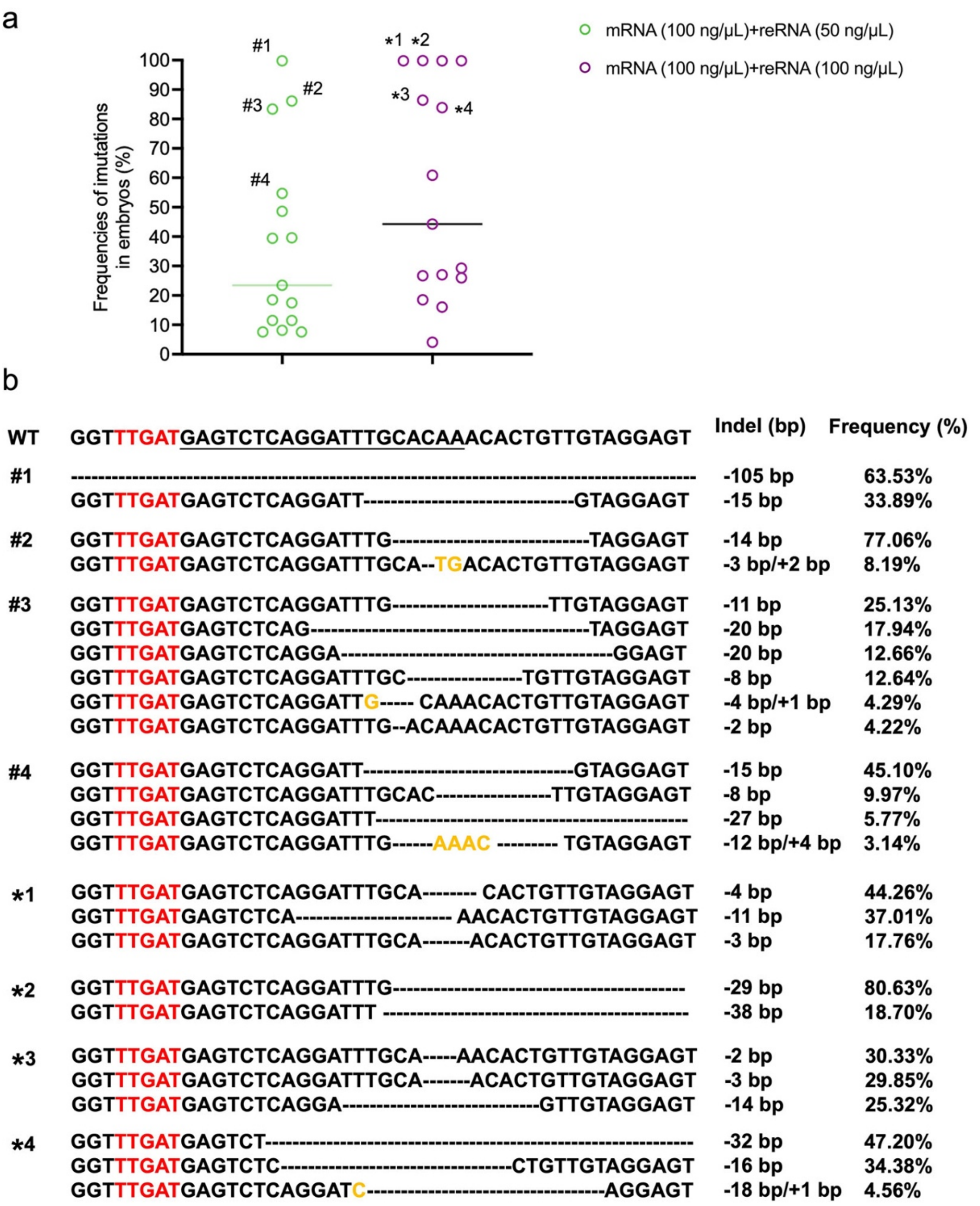

Supplementary Figure 8. TnpB379-mediated genome editing in mouse *Mstn* by microinjection.

(a) Indel frequency in mouse embryos generated by microinjection. PCR amplicons from the target regions in *Mstn* were analysed by targeted deep sequencing. Each dot represents one individual. **(b)** Targeted deep sequencing to analyse mutations at the target site. The TAM and target sequences are indicated in red and underlined, respectively. Microhomology sequences are shown in orange.

a

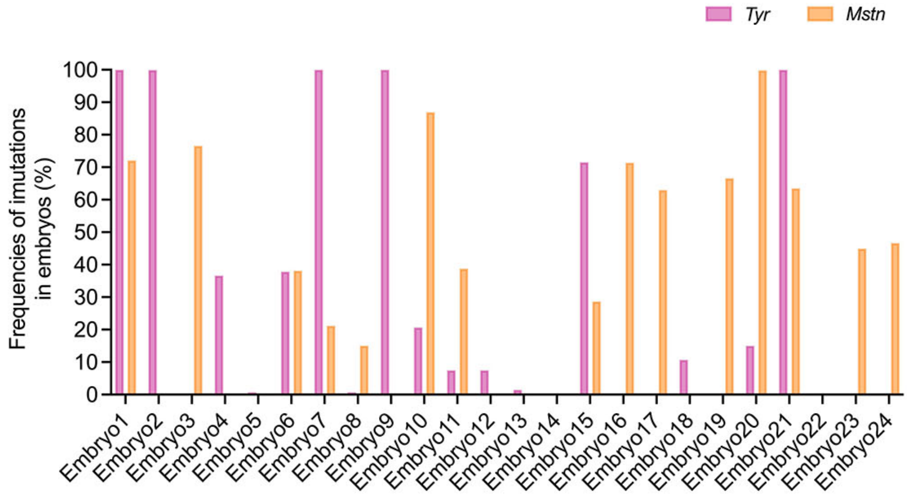

b

			Indel (bp)	Frequency (%)
**Embryo1**	***Tyr***			
		--TTCTTACC	-64 bp	85.40%
		TTTTTGATTTGAGTGTCTCCGAGATTAA------------------------------------	-16 bp/+6 bp	14.15%
	Mstn			
		GGTTTGATGAGTCTCTAAT-----------------------ACTGTTGTAGGAGT	-14 bp/+6 bp	31.57%
		GGTTTGATGAGTCT-------------------------------------TGATATA---------T	-27 bp/+7 bp	23.47%
		---	-105 bp	13.67%
		GGTTTGATGAGTCTCAGGATTTC----------------------------TAGGAGT	-14 bp	1.97%
**Embryo6**	***Tyr***			
		TTTTTGATTTGAGTGTCT-- TCTTACC	-20 bp	25.38%
		TTTTTGATTTGAGTGTCTCCGAAAA------ TAAGTTCTTTTCTTACC	-3 bp	7.82%
		TTTTTGATTTGAGTGTCTCCGAA--------------------TCTTTTCTTACC	-10 bp	3.74%
	Mstn			
		GGTTTGATGAGTCTCAGT----------------------CACTGTTGTAGGAGT	-12 bp/+1 bp	20.76%
		GGTTTGATGAGTCTCAGGATTTGCA --- AACACTGTTGTAGGAGT	-2 bp	13.65%
		GGTTTGATGAGTCTCAGGATTTGCACA --- CACTGTTGTAGGAGT	-2 bp	10.24%
		GGTTTGATGAGTCTCAGGATTTG--	-29 bp	3.93%
**Embryo6**	***Tyr***			
		TTTTTGATTTGAGTGTCTC--	-28 bp	99.42%
	Mstn			
		GGTTTGATGAGTCTCAGGATT --------------- CACTGTTGTAGGAGT	-8 bp	20.90%
		GGTTTGATGAGTCTCAGGATTTGCA----AACACTGTTGTAGGAGT	-2 bp	5.11%
		GGTTTGATGAGTCTCAGGATT------ACAAACACTGTTGTAGGAGT	-3 bp	3.29%
**Embryo10**	***Tyr***			
		TTTTTGATTTGAGTGTCTCCGAAAA--------------GTTCTTTTCTTACC	-6 bp	19.89%
		TTTTTGATTTGAGTGTCTCCGAAAAGAA------- GTTCTTTTCTTACC	-2 bp	2.15%
	Mstn			
		GGTTTGATGAGTCT---	-32 bp	47.20%
		GGTTTGATGAGTCTC -------------------------------CTGTTGTAGGAGT	-16 bp	34.38%
		GGTTTGATGAGTCTCAGGATTC-------------------------------AGGAGT	-18 bp/+1 bp	4.56%
**Embryo21**	***Tyr***			
		TTTTTGATTTGAGTGTCT---------------------------------------TTTCTTACC	-18 bp	62.13%
		TTTTTGATTTGAGTGT-------------------------------------TCTTTTCTTACC	-17 bp	36.77%
	Mstn			
		GGTTTGATGAGTCTCAGGA--GT	-23 bp	50.16%
		GGTTTGATGAGTCTCAGGATTC-------------ACACTGTTGAGGAGT	-7 bp/+1 bp	7.97%
		GGTTTGATGAGTCTCAGGAT---	-41 bp	2.32%
		GGTTTGATGAGTCTCAGGATTTGCA----- AACACTGTTGAGGAGT	-2 bp	1.38%
		GGTTTGATGAGTC------------------------------ ---CACTGTTGAGGAGT	-16 bp	1.07%

Supplementary Figure 9. TnpB379-mediated multigenome editing in mouse *Tyr/Mstn* by microinjection.

(a) Indel frequency in mice embryos with multigene editing generated by microinjection. PCR amplicons from the target regions in *Tyr* and *Mstn* were analysed by targeted deep sequencing. Light purple indicates the *Tyr* mutation, and light yellow indicates the *Mstn* mutation. **(b)** Representative indels information of mouse embryos with two gene mutations at the target site. The TAM and target sequences are indicated in red and underlined, respectively. Microhomology sequences are shown in orange.

Supplementary Materials and Methods

Ethics approval

This study was approved by the Ethics Committees of China Agricultural University (AW41213202-3-1).

Vector construction

IS200/IS605 ISDraTnpB[1], containing *tnpB* encoding the TnpB protein and reRNAs (right element RNA), was synthesized by Sangon Biotech (Shanghai, China) and cloned into the pX330 vector (Addgene 42230) under the CAG and U6 promoters, respectively (pCAG-ISDra2TnpB-U6-reRNAs). To construct TnpB expression vectors for gene editing, the pCAG-ISDra2TnpB-U6-reRNAs plasmid backbone was cut using *Bbs* I (R0539S, New England Biolabs, US), and a 20-bp target sequence next to the 5′-TTGAT transposon-associated motif (TAM) was synthesized, annealed and subcloned into the backbone. All vectors were confirmed through restriction digestion and Sanger sequencing. The TnpB and Cas9 target sequences (20-bp target) used in this study are listed in **Supplementary Table 1-2**.

Cell culture and transfection

Human HEK293T, HeLa, and mouse NIH/3T3 cells were obtained from the American Type Culture Collection (ATCC) and cultured in Dulbecco's Modified Eagle Medium (DMEM, Gibco, US), supplemented with 10% fetal bovine serum (FBS) (v/v) (Gibco, US) and incubated at 37°C with 5% CO_2. For plasmid transfection, cells were plated in a 12-well plate at a density of 2×10^5 cells/well and transfected at approximately 70% confluence using Lipofectamine™3000 (Invitrogen, US). Each well received 2 μg of plasmid encoding TnpB and its reRNA. Cells were collected 72 h after transfection for further analysis.

In vitro transcription

The *in vitro* TnpB transcription templates were amplified from the pCAG-ISDra2-TnpB-U6-reRNAs plasmid backbone using TnpB-up/down primers. TnpB mRNA was transcribed using mMESSAGE mMACHINE T7 ULTRA (Invitrogen, US) and purified with the MEGA clean Kit (Invitrogen, US). The *Tyr* or *Mstn* reRNA *in vitro* transcription templates was amplified from its expression vector using primers TYR-reRNA-F/R, transcribed using the MEGAshortscript Kit (Ambion, US) and purified by ethanol precipitation.

Zygote injection, embryo culture, and embryo transplantation

For mouse gene editing, superovulated female B6D2F1 (C57BL/6 × DBA2J) mice (7-8 weeks old) were mated to C57BL/6 males, and fertilized embryos were collected from oviducts. The TnpB expression vector (50 ng/μL) was injected into the pronuclei of fertilized eggs with well-defined pronuclei in a droplet of M2 medium (Sigma, US) using a FemtoJet microinjector (Eppendorf, DE) with a constant flow setting. The injected zygotes were cultured in M16 medium (Sigma, US) at 37 °C under 5% CO_2 in air. Subsequently, 25-30 embryos were transferred into the oviducts of surrogate ICR females.

***In vivo* AAV8.TnpB+reRNA delivery and liver tissue processing**

AAV8 for mouse injection was generated by PackGene Biotech. For the AAV8 vector injections, 6-week-old male C57BL/6NJ mice were injected with 4 $x10^{11}$ or 8 $x10^{11}$ genome copies per mouse via the tail vein, targeting either *Pcsk9* or *Rosa26* with validated sgRNA. Blood (~ 200 μL) was collected from the facial vein at 0, 14, and 28 days after vector administration. Serum was isolated using a serum separator (BD, Cat. No. 365967, US) and stored at -80 °C until assay. Mice were sacrificed 28 days after vector administration, and liver tissues were collected for analysis. Serum Pcsk9 levels were measured by the Mouse Proprotein Convertase 9/Pcsk9 Quantikine ELISA Kit (R&D Systems, cat. no. MPC900, US) according to the manufacturer's instructions. Serum LDL levels were measured by the InfinityTM colorimetric endpoint assay (Thermo-Scientific, US) following the manufacturer's protocol. Liver tissue fractions were ground and resuspended in 150 μL of RIPA lysis buffer for anti-Pcsk9 Western blotting. Protein levels were determined with the Pierce BCA Protein Assay Kit (Thermo Fisher Scientific, US), and equal amounts of proteins were separated by SDS–PAGE electrophoresis followed by transfer to nitrocellulose membranes (Millipore, US). Membranes were incubated with goat anti-mouse-Pcsk9 (1:10,000, cat. no. AF3985-SP, R&D Systems, US) and rabbit anti-GAPDH (1:5,000, cat. no. 4970, Abcam, UK). Horseradish peroxidase (HRP)-conjugated anti-mouse or anti-rabbit IgG secondary antibody (dilution, 1:10000; Sino-American Co., Beijing, China) was used, and the signal was revealed by enhanced chemiluminescence substrate (Thermo Fisher Scientific, US).

Indel characterization

Genomic DNA was extracted from transfected cells and mouse tissues using DNeasy Blood & Tissue Kits (QIAGEN, DE). On-target sites were amplified from genomic DNA using Q5 DNA Polymerase (NEB, US) with the primers listed in **Supplementary Table S3**. The PCR setting consisted of initial denaturation at 98 °C for 30 s; 35 cycles of 98 °C for 15 s, 56-60 °C for 15 s, and 72 °C for 15 s; and a final incubation at 72 °C for 5 min. The resulting amplicons were cleaned using the AxyPrep™ DNA Gel Extraction Kit (AXYGEN, US), and paired-end sequencing of PCR amplicons was performed on the Illumina Nextseq 500 (2 × 150) platform at Sangon Biotech (Shanghai, China). Insertion or deletion mutations (INDELs) were analyzed using CRISPResso2 [2].

Off-target analysis with in silico prediction

To evaluate the specificity of TnpB, TnpB379, Cas9, the CRISPR RGEN Tools (Cas-OFFinder, http://www.rgenome.net/cas-offinder/) was used to predict the potential off-target sites. To predict potential off-target sites of Cas9, search queries were 20-nt on-target Cas9 spacer sequences. PAM type was set to 'NGG' and the mismatches were set to 3 and the DNA bulges were set to 1. For TnpB and TnpB379, search queries were 20-nt on-target TnpB/TnpB379 spacer sequence. The PAM of research was set to 'TTGAT' and the mismatches were set to 5. All other parameters were left as default. Off-target sites of each group were selected manually in order from the lower mismatches number to higher mismatches number. Off-target sites of each guide sequence were selected in order from the lower mismatches number to higher mismatches number. The predicted potential off-target sites with one or more mismatches were selected to design primers using the online Primer3web (https://primer3.ut.ee). The predicted potential off-target sequences of the top 5-10 designable primers on the list were amplified and sequenced to evaluate the specificity of TnpB, TnpB379, Cas9. All predicted potential off-target sites and primers used in off-target analysis are provided in **Supplementary Table 4**.

Whole genome sequencing

Mutant mice or cells were subject to whole genome sequencing (WGS). To construct the WGS library, 1 μg of genomic DNA was fragmented to around 300 bp by ultrasonication using a Covaris S2 system. Then, the sheared DNA fragments were used for library construction and the library were sequenced using Hiseq X Ten platform (Illumina) at Sangon Biotech (Shanghai, China). The WGS data of wild-type mice or cells have the same genetic background with the

founders was used in this study. The analysis process was conducted as described previously[3].

Supplementary Tables

Supplementary Table 1 The TnpB target sequences (20-bp target) used in human and mouse cells in this study.

Target gene	Target Sequence
Human EMX1-1	GTGATGGGAGCCCTTCTTCT
Human AGBL1-1	TGTTGGCTCAAACACCAGAT
Human AGBL1-2	AATGAATGGCTACTCTAACC
Human ROSA26-1	CTTGGGTAGCCTGGTGTCTC
Human ROSA26-2	TTTCATTTTATCCTTTTTCC
Human ROSA26-3	CCATCTTACATCTTGTTAGG
Human AAVS1-1	TCCCGGCCAGTTTCTCCACC
Human AAVS1-2	AAACCCACGTGGGGTACCCT
Human CCR5	GTCATAGATTGGACTTGACA
Human DNMT3B	GTTAGGATTAGGTGGGCTCA
Human DNMT1	GCCAACGAGTCTGGCTTTGA
Human CDKN2A	AATATATCCATTTGATAATA
Human RUNX1	GGCTCTGTGGTAGGTGGCGA
Mice Tyr-1	TAGAAGAAACATTTTTGATT
Mice Tyr-2	TTGAGTGTCTCCGAAAAGAA
Mice Ttyr-3	ATCATTAAACATGGGTGTTG
Mice Rosa26-1	AAGGCTGCAGAAGGAGCGGG
Mice Rosa26-2	CCTTTGCCTTGATCCTTAAT
Mice Rosa 6-3	TACTGCTTACTAAAATTTTG
Mice H11-1	ACTTGCCACAAGCTACTTTC
Mice H11-2	TCCTTGATACTTGCCACAAG
Mice H11-3	TTTGTAGAGTATAATATTGA
Mice Oct4-1	CTTTTGCCCTTCTGGCGCCG
Mice Oct4-2	CAGGAGCCTGGCCTGTCTGT

Mice Tet1	GTCTTCGTCTTCATCTGGAT
Mice Tet2	GCTTCCAGAACAGCATCAGA
Mice Tet3	CTGAATGGGCTTTCGGACAC
Mice Mstn-1	GAGTCTCAGGATTTGCACAA
Mice Mstn-2	TTCAATGCCTAAGTTGGATT
Mice Polq	TCCAAAAGTGGTACAGGGCG
Mice Pcsk9	GACATAGCCCCGGCGGGCAG

Supplementary Table 2 The Cas9 target sequences (20-bp target) used in human cells in this study.

Target gene	Target Sequence
Human EMX1-1	GAGTCCGAGCAGAAGAAGAA
Human AGBL1-1	TTGGCTCAAACACCAGATTT
Human ROSA26-1	GTCTGGTTTCGCGAGACACC
Human CCR5	TAATAATTGATGTCATAGAT
Human DNMT3B	GTTAGGATTAGGTGGGCTCA
Human DNMT1	GTCTGGCTTTGAGAGTTATG
Human CDKN2A	CAAATGGATATATTATCAAA
Human RUNX1	CTGTGGTAGGTGGCGACTTG

Supplementary Table 3 Primers used for genomic DNA amplification.

Primer	Sequence
Human EMX1-1-F	cccctaaccctatgtagcct
Human EMX1-1-R	cagggagtggccagagtccag
Human AGBL1-F	ctgacccatcaggtggtttt
Human AGBL1-R	agagatgctgcaaagccatt
Human ROSA26-1-F	ccctaccaggcatcttagca
Human ROSA26-1-R	tggactgtgaagacggtcag
Human ROSA26-2-F	tcttgggtagcctggtgtct
Human ROSA26-2-R	cagagacagcaatttggaagc

Human ROSA26-3-F	cgtcttcacagtccaggtga
Human ROSA26-3-R	aatttgtgttgggtcgcatt
Human AAVS1-F	gtgtggggtgaccagcttat
Human AAVS1-R	cagcccctcctactctagcc
Human CCR5-F	tgcttggccaaaaagagagt
Human CCR5-R	gctcttcagccttttgcagt
Human DNMT3B-F	tctgagcaaaaccaacccaa
Human DNMT3B-R	gactacaggcaatggaccac
Human RUNX1-F	aaagccaaaattccgggagt
Human RUNX1-R	tcagtgcacagaaacaagct
Human DNMT1-F	ccctgacctcaaatatgggc
Human DNMT1-R	tcgaactcctgacctgaagt
Human CDKN2A-F	tggagacgggagaagaacat
Human CDKN2A-R	gaagtccttcgtcttggtca
Mice Tyr-1/2-F	gcaccatctggacctcagtt
Mice Tyr-1/2-R	atgggtgttgacccattgtt
Mice Tyr-3-F	gggcccaaattgtacagaga
Mice Tyr-3-R	cagaaaccctggtgcttcat
Mice Rosa26-1-F	gacatgctcacccgagtttt
Mice Rosa26-1-R	ggatcaaggcaaaggatcaa
Mice Rosa26-2-F	caccacaaatcgaggctgta
Mice Rosa26-2-R	tattgggggaggagacatcc
Mice Rosa26-3-F	gcttaactttccactcccatga
Mice Rosa26-3-R	aaaaatacaagctcacaagacctta
Mice H11-F	agcagaggcaagggaaagac
Mice H11-R	atatgccacgtgtgtgaacg
Mice Oct4-1-F	ccaatcagcttgggctagag
Mice Oct4-1-R	ggcagaggaaaggatacagc
Mice Oct4-2-F	caaggcaagggaggtagaca
Mice Oct4-2-R	gatcacctggggtttgagaa

Mice Tet1-1-F	tttgcaaaggaccaatgaca
Mice Tet1-1-R	tcgccagctaagagaggttc
Mice Tet1-2/3-F	gaaggggagagccaagagat
Mice Tet1-2/3-R	atcaagaggcatgcaggttt
Mice Tet2-1-F	gtttctgcccattctgagga
Mice Tet2-1-R	catgcttccttggatggttt
Mice Tet2-2/3-F	ctccccgtcttgttcttcag
Mice Tet2-2/3-R	gaatctcttgctccctgtgc
Mice Tet3-1-F	ctctgagggcagtgaagacc
Mice Tet3-1-R	aggtggaatgagaaggctga
Mice Tet3-2-F	acagcccttcagcaacatct
Mice Tet3-2-R	ttctgaggcttggggtttta
Mice Tet3-3-F	caaaccacccaaggaaaaga
Mice Tet3-3-R	gcaaatagcgcaagagaagg
Mice Mstn-1-F	gggcctgatgtcagtca
Mice Mstn-1-R	ctggtcctgggaaggttaca
Mice Mstn-2-F	cccgtcaagactcctacaaca
Mice Mstn-2-R	ctggtcctgggaaggttaca
Mice Mstn-3-F	cccgtcaagactcctacaaca
Mice Mstn-3-R	gtctggcttatgagcatagga
Mice Polq-1-F	aatgctgtgcagattgttgg
Mice Polq-1-R	tccttctcactctcctcatcaa
Mice Polq-2/3-F	gatgttttggagatatgactgacg
Mice Polq-2/3-R	tcaccacacagtgacagtacca
Mice Pcsk9-F	cctcacgttctctgcttctc
Mice Pcsk9-R	gcacactaaggtcagtcgag

Supplementary Table 4 The predicted potential off-target sites of TnpB or TnpB379 and primers used in off-target analysis.

Target gene	The potential off-target sequence	Primer sequence

Human EMX1-OT1	cTGATGGGAGCCCtTtTTCt	ACAAATGAATACGTTGTGGAA
		TAGACTAGCAGCCCTCT
Human EMX1-OT2	GTctgGGGAGgCCTgCTTCT	TTTTGTGGTACTGGGCTGC
		TGCCATCATCCATTGAGCATC
Human EMX1-OT3	cTGgTGGGAtCtCaTCTTCT	TCATGGGGTCTTGGCTTTCA
		AGAGTCGCCTCTAAGGGTTG
Human EMX1-OT4	GTGATatGAtCCaTTCTTtT	ACATCAGTCTCTTGACCCAGC
		ACCTCTGAGAGATTCTGAACAA
Human EMX1-OT5	cTGcTtGGAGCCCTTCcTgT	AAAGCCATTCTAGCACAG
		ATTGATAATTTTCATCGATTC
Human EMX1-OT6	GTtATtGaAcCCCTTCTaCT	AACTCTCATACACTTAAGCTG
		TCTACATGGTGCAGTATGTC
Human EMX1-OT7	aaGATctGAGCCCTTtTTCT	TTGCTCATGGAAATGAACAGG
		AGAGAGAAACAATAGAAGCCT
Human EMX1-OT8	GacATGGGAGgCaTTaTTCT	AGAGAGAAACAATAGAAGCCT
		TTGTCAATAAAAACGGACTTA
Human AGBL1-OT1	TtTTGGgTCAgACACCAaAT	CCGTGAGAACCACGTGAAGT
		CTCAAGCAGTGTCTCATGCAA
Human AGBL1-OT2	TGTTtGtgCAAACACgAGAT	TTCACTGCTCTGAAGGACACAC
		CTGTTTTTCCCTTGCAATTGTCC
Human AGBL1-OT3	TcTTGGCTCAAAaAaCAGAg	GGGCTGCCCAGGAACTAAAA
		ATTCAGCCCAGGATCCCTCT
Human AGBL1-OT4	TaTTGGaTtAAcCACCAGAg	TCCACACCTCCTCTGTGTAT
		TCTCAGCAAACAAAGGACAAAG
Human AGBL1-OT5	TcTTGGCTCAttCAaCAaAT	AACGCATCACTTGACTGGCA
		TCCCAGAGATTTTCACTGAGTTT
Human AGBL1-OT6	TcTTGGCTTaAAtACCActT	AATACTGCAATCAGCGTGGC
		CCCTTGTGGAATTTGCAGCC
Human	TtTTGGgTCActCACCAGtT	CCTCTCCCTAATCACCC

AGBL1-OT7		CCCACATTTCTCAACACATGA
Human AGBL1-OT8	TGTTGGgTgAAACAatAGAa	TGCTATCTCCAGGTAAAC<br>ATATAGTGCTTTAAGATACCA
Human ROSA26-OT1	CTTaGaTAGCCTGGTGaCTa	GATATGAAATTATTAGTTGGA<br>GTAAAGTGACCAAACATACAA
Human ROSA26-OT2	tTTGGGTAGCaTGaTGTgTC	GCTCATTTTCGTCAGGTT<br>CAAACAAATGGAAGAACATTC
Human ROSA26-OT3	gTTaGGTAGCaTGaTGTCTC	GCCCAAAGCAAGTTATAGATT<br>TTTCTGCACATAGCTAGCCAG
Human ROSA26-OT4	gTTGGaTAGCaTGaTGcCTC	GGGGAATTCCTCATTGCCTA<br>GGAAAAACGTCCCATGCCTAT
Human ROSA26-OT5	CTTGtGTAcCCTGcTGgCTa	TGGTCCAGAACACTTCTAGGATT<br>TTGCCTTGACAATCGGGGAA
Human ROSA26-OT6	CTTGcacAGCCTGGTGaCTa	GCCCCCAATCTCTTCTGACT<br>AGGAAACAACCTGGAACACAG
Human ROSA26-OT7	CTTGtGTAtCaTGtTGTCTg	TTGACTAAATGAAATGGACCT<br>GGCTGTTCTTGAACTCCTAAC
Human ROSA26-OT8	gTTGGcTgGgCTGGTGTCTa	AGTCTGGTCAGTCAAGCTGC<br>GGCCTGTTTTTGAACAGGGTT
Human DNMT1-OT1	tCCAACcAaTCTGGgTTTGA	CCAGAGAGGCAGCTCTGTTT<br>CCAGGACTCAAGAGGCTGTG
Human DNMT1-OT2	CAtgGtGTCTGGgTcTGA	CAGAAATAGATAAGATGCAG<br>CGAAAACAATTTTAGTTGATA
Human DNMT1-OT3	tCCAAgttGTgTGGCTTTGA	ACGTCAACAATGGCAGTCCT<br>GAACCCCCTGGGTCTCAGTA
Human DNMT1-OT4	GaCAttaAGTCTGaCTTTGA	GTGTTAGCATGGTATAACTTC<br>ACCTGTCAGAGGGTTG
Human DNMT1-OT5	tCCAAacAGTCTGGCTTacA	GCCAACATTCAAATTCAG<br>TCCATTGAATATTTATCATTG

Human DNMT1-OT6	GCCAACaAGTCTatCTTaGg	ACAAACACTTAACCATTCATA
		TGAGACCAAGAAATACTTATC
Human DNMT1-OT7	tCCtACcAGaCTGGgTTTGA	TAGCCCACTTGGGTCTCTCT
		ATCCCTGCTGATGCCAATCC
Human DNMT1-OT8	GatAAgGAGTgTGGCTTTGc	GGGGTACAGACACTTGAG
		TGGAAGCTTCAAACACTATCA
Human CCR5-OT1	cTCATAtATaGGACTTGtCA	GAAAATGTTTCCCTTTCATCT
		GAAAATGTTTCCCTTTCATCT
Human CCR5-OT2	GaCAgAGATTGGACTgtACA	TGACTCACAAAACTTTGGCTTCT
		GCACGTTTCTCTTCCTTATGACTT
Human CCR5-OT3	GTgAatGAgTGGACTTGgCA	TTTGGAGAAGGAGAACCCGC
		CTCCGGTCAGTATGGAAGCC
Human CCR5-OT4	tTtATAGATTGGAaTTtAtA	TTAGGGCTTTTGCGTGAGGT
		TTCTCCCTCTACCCTTCAGCA
Human CCR5-OT5	GTaAaAGATTGGAaaTaACA	GTGCTGGGATTACAGG
		CCAAGACTCAAAATCTGTTCT
Human CCR5-OT6	GTCATAaATctGACTTcAtA	CCAAGCAATTGTCAGGTATAA
		GGGTTCAAGCAGTCCTC
Human CCR5-OT7	GTCATAGAaTaaACTaGAaA	GCTAAATGACAAGTTAATGGG
		AGACAAATGATGAATTGGGA
Human CCR5-OT8	tTtATAGATTGGAtTTcAaA	TGCTGTCTATTGAGAGGTGTA
		GTTTCTGTAAAGAACCTTGA
Mice Rosa26-OT1	AAaGCTcCAGAAGGtGaGGG	TTTTGCAAAGAAGAGAAACGA
		CACGCACCCATGAATACACA
Mice Rosa26-OT2	AAGGaTGCtGAcGGAGgGGa	AAGAGCATTCCTCCTATT
		TGCTGTTTTTGTTCCTATGTC
Mice Rosa26-OT3	AAGcCcaCtGAAGGAGCGtG	GGCCTTTTCCAAACTT
		TGTGATTGCCTGTGAACCTAC
Mice	AAGaCTGaAGAtGaAGCaGG	GTTAACAAAGGAGCTTACTTA

Rosa26-OT4		TTCTCTTTTGCATAGGTAACT
Mice Rosa26-OT5	tgGGCaGCAGAAGGAGgGaG	CTACTATTTATGGGGTCT
		CACACCTAGGTGGGATAAATG
Mice Pcsk9-OT1	GACATAGCCatacCtGGCAG	CTGAGCTGTCTCTCACCATTA
		TTGCTTCTCAAGAGCC
Mice Pcsk9-OT2	aACATAaCagCGGaGGGCAG	ACTCTGTGTGGCTCATTGCCC
		CACCTCCCATTTTCCGCTT
Mice Pcsk9-OT3	tACATAGCCCaGGCtGGCct	GGAGCTGACTAACTCGG
		GAATATGCTCAAACCTATTGC
Mice Pcsk9-OT4	ctCATAGCCCCaGCtGGaAG	AAGCCGGGCATTTATCA
		GAGTTCTTTGAGCCCCTAGTC
Mice Pcsk9-OT5	tACATAGCCCtGGCtGGCct	CAACAGGGAACTCCATAAACC
		GCAGGGAAGTAAATTGCCTTA

References

1 Karvelis, T. *et al. Nature* **599**, 692-696 (2021).

2 Clement, K. *et al. Nat.Biotechnol.* **37**, 224-226 (2019).

3 Liu, Y. *et al. Cell Discov.* **6**, 27 (2020).
